# Brain imaging traits and epilepsy: Unraveling causal links via mendelian randomization

**DOI:** 10.1002/brb3.70051

**Published:** 2024-09-30

**Authors:** Fangyan Li, Maowen Tang, Cheng Hao, Menghua Yang, Yue Pan, Pinggui Lei

**Affiliations:** ^1^ Department of Radiology Affiliated Hospital of Guizhou Medical University Guiyang Guizhou China

**Keywords:** epilepsy, imaging‐derived phenotypes, magnetic resonance imaging, Mendelian randomization

## Abstract

**Background:**

Epilepsy, a complex neurological disorder, is closely linked with structural and functional irregularities in the brain. However, the causal relationship between brain imaging‐derived phenotypes (IDPs) and epilepsy remains unclear. This study aimed to investigate this relationship by employing a two‐sample bidirectional Mendelian randomization (MR) approach.

**Methods:**

The analysis involved 3935 cerebral IDPs from the UK Biobank and all documented cases of epilepsy (all epilepsies) cohorts from the International League Against Epilepsy, with further validation through replication and meta‐analyses using epilepsy Genome‐Wide Association Studies datasets from the FinnGen database. Additionally, a multivariate MR analysis framework was utilized to assess the direct impact of IDPs on all epilepsies. Furthermore, we performed a bidirectional MR analysis to investigate the relationship between the IDPs identified in all epilepsies and the 15 specific subtypes of epilepsy.

**Results:**

The study identified significant causal links between four IDPs and epilepsy risk. Decreased fractional anisotropy in the left inferior longitudinal fasciculus was associated with a higher risk of epilepsy (odds ratio [OR]: 0.89, *p* = 3.31×10^−5^). Conversely, increased mean L1 in the left posterior thalamic radiation (PTR) was independently associated with a heightened epilepsy risk (OR: 1.14, *p* = 4.72×10^−5^). Elevated L3 in the left cingulate gyrus was also linked to an increased risk (OR: 1.09, *p* = .03), while decreased intracellular volume fraction in the corpus callosum was correlated with higher epilepsy risk (OR: 0.94, *p* = 1.15×10^−4^). Subtype analysis revealed that three of these IDPs are primarily associated with focal epilepsy (FE). Notably, increased L1 in the left PTR was linked to an elevated risk of hippocampal sclerosis (HS) and lesion‐negative FE, whereas elevated L3 in the left cingulate gyrus was associated with HS‐related FE.

**Conclusions:**

Our research offers genetic evidence for a causal link between brain IDPs and epilepsy. These results enhance our understanding of the structural brain changes associated with the onset and progression of epilepsy.

## Graphical abstract

Study Design and Key Findings on Brain Imaging Traits and Epilepsy

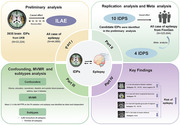

## 1 INTRODUCTION

Epilepsy, a prevalent neurological disorder impacting over 70 million individuals globally, presents significant challenges related to quality of life, morbidity, and the potential for premature mortality, particularly among those with persistent seizures (Thijs et al., 2019). These seizures are temporary occurrences characterized by abnormal, excessive, or synchronized neuronal activity in the brain, leading to observable signs and symptoms (Fisher et al., 2005). Epilepsies stem from various causes, such as structural abnormalities in the brain, as well as genetic variations, both monogenic and polygenic (Symonds et al., 2021). Overall, epilepsy represents a substantial public health challenge, imposing a considerable burden on both the globe and society as a whole.

Magnetic resonance imaging (MRI) is pivotal in the diagnostic and evaluative processes for epilepsy patients (Lapalme‐Remis & Nguyen, 2022). Imaging‐derived phenotypes (IDPs) are quantitative attributes derived from raw imaging data through processing and analysis, resulting in metrics that are interpretable and informative (Elliott et al., 2018; Gong et al., 2021). IDPs include detailed measures such as gray matter volume, surface area, and cortical thickness, as well as comprehensive data on white matter integrity and functional connectivity (Elliott et al., 2018). IDPs offer detailed visualization of epilepsy‐related abnormalities, including disruptions in white matter structural networks, neuronal migration disorders, neocortical sclerosis, and mesial temporal lobe sclerosis (Ai et al., 2024; Jack, 1996). Additionally, IDPs function as intermediate endophenotypes that link these structural abnormalities to genetic factors (Elliott et al., 2018). This integration enhances our understanding of the genetic underpinnings of epilepsy, refines diagnostic accuracy, and facilitates the development of personalized treatment strategies.

Numerous observational studies have explored the relationship between IDPs and epilepsy. For example, Keller et al. (2008) found significant volume reductions in 26 different brain regions in patients with temporal lobe epilepsy (TLE) compared to healthy controls, with the most pronounced reduction observed in the hippocampus. Similarly, Whelan et al. (2018) analyzed brain MRI data from 1727 healthy controls and 2149 epilepsy patients across 24 research centers worldwide, identifying structural changes in various brain regions associated with different forms of epilepsy. Beyond macrostructural changes in the brain, increasing evidence suggests that epilepsy may be linked to disruptions in brain network function, potentially arising from deficits in microstructural or functional characteristics (Braakman et al., 2012; **M**.**Wang et al**., 2023). Modern neuroimaging technologies, such as functional magnetic resonance imaging (fMRI) and diffusion tensor imaging (DTI), have significantly advanced the diagnosis of epilepsy (Ostrowski et al., 2023; Zhu et al., 2022). These techniques provide critical insights into the microstructural dynamics of white matter tracts and changes in functional connectivity within specific brain regions. For instance, Ai et al. (2024) used DTI to analyze the structural networks of 18 TLE patients and 29 age‐ and sex‐matched controls, revealing disruptions in the white matter structural networks of TLE patients. Similarly, Y. Zhang et al. (2023) examined DTI parameters before and after surgery in patients with medial temporal lobe epilepsy, identifying postoperative changes in several brain regions, including the inferior longitudinal fasciculus (ILF) and the cingulate gyrus. Despite these advancements, establishing a causal relationship between IDPs and epilepsy remains challenging due to potential residual confounding factors inherent in traditional observational studies (Yu et al., 2023).

Mendelian randomization (MR) studies utilize genetic variants associated with different exposures to investigate their potential causal associations with health outcomes, with the goal of reducing confounding and reverse causation biases (Skrivankova et al., 2021). Until now, this method has not been used to investigate the connection between IDPs and epilepsy. For our preliminary inquiry, we employed a bidirectional two‐sample MR analysis to evaluate the causal relationship between 3935 internally displaced persons IDPs and all documented cases of epilepsy (all epilepsies). Our analysis was based on data obtained from the International League Against Epilepsy (ILAE) (International League Against Epilepsy Consortium on Complex, Epilepsies, 2018). To assess the validity and reproducibility of our findings, we conducted validation and meta‐analysis on the FinnGen epilepsy cohort. Through the utilization of multivariable MR (MVMR), we were able to conduct a more in‐depth examination and pinpoint the specific independent determinants of epilepsy. Furthermore, we investigated the relationship between the identified IDPs and various subtypes of epilepsy. The findings of our study offer useful insights that have the potential to impact the development of early prevention, diagnostic techniques, and treatment methods for epilepsy, specifically in relation to imaging.

## 2 METHODS

### 2.1 Study design

**FIGURE 1:**
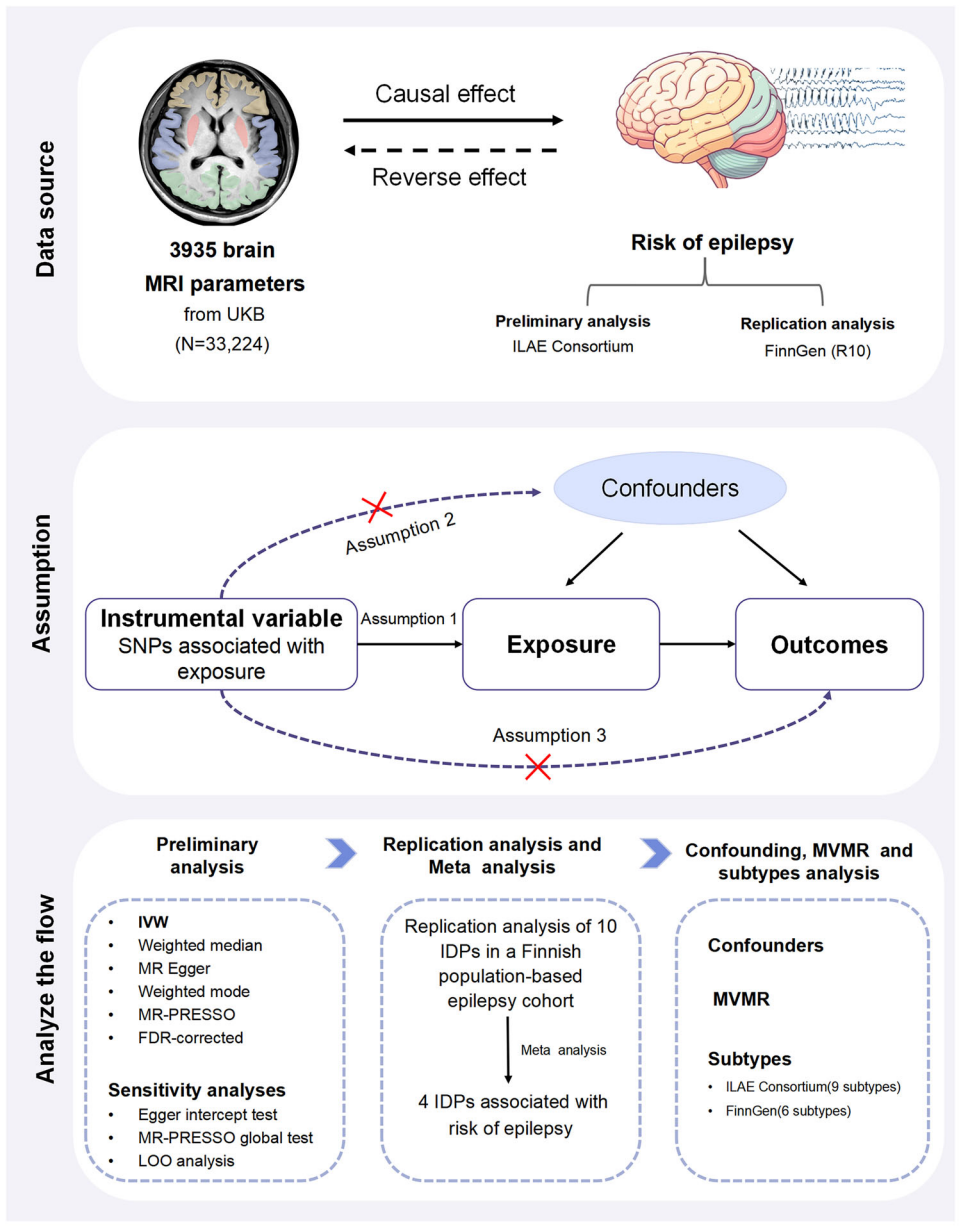
Flowchart illustrating the framework for Mendelian randomization (MR) analysis concerning imaging‐derived phenotypes (IDPs) and epilepsy. Assumption 1, the genetic variants have a strong association with the exposure; Assumption 2, these variants are not linked to any potential confounders affecting the exposure–outcome relationship; Assumption 3, the variants influence the outcome solely through their interaction with the exposure. FDR, false discovery rate; IDPs, imaging‐derived phenotypes; ILAE, International League Against Epilepsy; IVW, inverse‐variance weighting; LOO, leave‐one‐out; MVMR, multivariate Mendelian randomization; PRESSO, pleiotropy RESidual Sum and Outlier; SNPs, single nucleotide polymorphism; UKB, UK Biobank.

The study's design is depicted in Figure 1. The data employed in our analysis are publicly available, with the ethical considerations of the original study receiving approval from the relevant local ethics committee. Furthermore, this study adheres to the established Mendelian Randomization guidelines for reporting within the context of epidemiological observational studies (Skrivankova et al., 2021). We conducted all analyses using R Software version 4.3.3, which is accessible at https://www.R‐project.org. Specifically, we employed the TwoSampleMR (version 0.5.11), MendelianRandomization (version 0.8.0), MR pleiotropy RESidual sum and outlier (MR‐PRESSO) (version 1.0), meta (version 6.5.0), and MVMR (version 0.4) packages. All software packages utilized in this study are publicly available. We employed ChiPlot (https://www.chiplot.online/) to generate the heatmap, accessed on April 29, 2024.

### 2.2 Data sources of brain IDPs and epilepsy

Summary statistics for all 3935 brain IDPs were sourced from the Genome‐Wide Association Studies (GWAS) study by Smith et al. (2021), involving a dataset of 33,224 individuals of European descent, sourced from the 2020 UK Biobank release. These IDPs originated from three types of measurements: structural MRI, diffusion MRI (dMRI), and fMRI summary metrics (Smith et al., 2021). The nomenclature for the brain IDPs remained consistent with the original terminology.

The preliminary investigation utilized epilepsy‐related GWAS summary statistics (GWAS ID: ieu‐b‐8, all documented cases) from the 2018 ILAE Consortium on Complex Epilepsies (International League Against Epilepsy Consortium on Complex, Epilepsies, 2018). This cohort included 15,212 epilepsy cases and 29,677 control subjects (Table 1). Notably, about 95.5% of the participants in the study were of European descent. For the replication analysis, the epilepsy GWAS dataset (Phenocode: G6_EPILEPSY) was obtained from the 10th release of FinnGen, a database containing genotype and health registry information from Finnish volunteers. The dataset includes a cohort consisting of 12,891 individuals with epilepsy and 312,803 control participants. In these two cohorts, epilepsy encompasses all documented cases, including generalized epilepsy (GE), focal epilepsy (FE), and so on, collectively referred to as “all epilepsies.” The GWAS data for subtype analysis primarily originate from the 2018 ILAE Consortium on Complex Epilepsies, which includes nine subtypes (International League Against Epilepsy Consortium on Complex, Epilepsies, 2018), and thefinnGen Database (R10 version), encompassing six subtypes. These subtypes include GE, FE, and others. Detailed information is provided in Table 1.

**TABLE 1:** Information for the Genome‐Wide Association Studies (GWAS) summary data used in this study.

ID	Trait	Abbreviation	Consortium	Sample_N	Cases_N	Control_N
25001‐27772	Brain imaging‐derived phenotypes	IDP	UK Biobank	33224	–	–
ieu‐b‐8	Epilepsy, all documented cases	–	ILAECCE	44889	15212	29677
ieu‐b‐10	Focal epilepsy, all documented cases	FE	ILAECCE	39348	9671	29677
ieu‐b‐15	Focal epilepsy, documented lesion other than hippocampal sclerosis	–	ILAECCE	32747	3070	29677
ieu‐b‐12	Juvenile absence epilepsy	–	ILAECCE	30092	415	29677
ieu‐b‐16	Generalized epilepsy with tonic‐clonic seizures	–	ILAECCE	29905	228	29677
ieu‐b‐17	Juvenile myoclonic epilepsy	–	ILAECCE	30858	1181	29677
ieu‐b‐11	Focal epilepsy, documented lesion negative	–	ILAECCE	32393	2716	29677
ieu‐b‐14	Focal epilepsy, documented hippocampal sclerosis	HS‐FE	ILAECCE	30480	803	29677
ieu‐b‐9	Generalized epilepsy, all documented cases	GE	ILAECCE	33446	3769	29677
ieu‐b‐13	Childhood absence epilepsy	–	ILAECCE	30470	793	29677
G6 EPLEPSY (R10)	Epilepsy	–	FinnGen	325694	12891	312803
FE (R10)	Focal epilepsy	FE	FinnGen	406816	7526	399290
FE_MODE (R10)	Focal epilepsy, mode (most common among epilepsy diagnosis)	–	FinnGen	400680	1390	399290
FE_STRICT (R10)	Focal epilepsy, strict definition	–	FinnGen	406125	6838	399287
GE (R10)	Generalized epilepsy	GE	FinnGen	400700	1413	399287
GE_MODE (R10)	Generalized epilepsy, mode (most common among epilepsy diagnosis)	–	FinnGen	401730	2440	399290
GE_STRICT (R10)	Generalized epilepsy, strict definition	–	FinnGen	400012	725	399287

Abbreviations: FE, focal epilepsy; GE, generalized epilepsy; HS‐FE, hippocampal sclerosis‐related focal epilepsy; IDP, imaging‐derived phenotype; ILAECCE, The ILAE Consortium on Complex Epilepsies.

### 2.3 IVs selection

In MR studies, genetic variants used as instrumental variables (IVs) are often single nucleotide polymorphisms (SNPs) (Skrivankova et al., 2021). When conducting MR, three core assumptions were taken into account (Figure 1): first, the genetic variants have a strong association with the exposure; second, these genetic variants are not linked to any potential confounders affecting the exposure–outcome relationship; and third, the genetic variants influence the outcome solely through their interaction with the exposure (Xia et al., 2023). When conducting MR analyses of IDPs and all epilepsies, we addressed the first assumption by modifying the selection criteria to a significance threshold of *p* < 5 × 10^−6^ due to the limited availability of suitable IVs (Jiang et al., 2024). A thorough selection process for independent SNPs as IVs was then undertaken. This involved considering the linkage disequilibrium (LD) coefficient (*r* ^2^ = 0.001) based on the European 1000 Genomes Project panel within a 10,000‐kb window to eliminate palindromic SNPs (Jiang et al., 2024). In order to evaluate the adequacy of “weak instruments,” individual and aggregate *F*‐statistics were computed for the sets of instrumental variables linked to IDPs and epilepsy. Instruments that obtained an *F*‐statistic greater than 10 were deemed to be robust. Those whose *F*‐statistics were less than 10 were omitted and reanalyzed thereafter. Additionally, MR Steiger filtering was employed to verify whether the estimates from the MR analysis accurately reflected the true causal direction (Hemani et al., 2017). This process led to the identification of 18,943 SNPs associated with IDPs, which were used for subsequent causal inference analysis (see Table S1). We employed identical screening criteria for IVs associated with exposure when conducting the reverse (Table S3), replication studies (Table S6), and subtypes MR studies.

### 2.4 Preliminary analyses of MR and sensitivity assessments

In this study, we performed two‐sample MR to investigate the potential causal links between the incidence of IDPs and epilepsy. The major method for determining causation was inverse‐variance weighting (IVW) regression using a multiplicative random effects framework. The IVW approach gives high‐power findings assuming that all IVs are authentic (Burgess et al., 2013). Three more MR techniques were applied to increase the validity of our findings. Regardless of the reliability of IVs, the MR–Egger technique gives a dependable approximation by computing causal effects using Egger regression's slope coefficient (Bowden et al., 2015). In contrast, the weighted median technique protects against invalid IVs up to a 50% threshold (Bowden et al., 2016). Furthermore, the weighted mode technique is well known for controlling bias and reducing the likelihood of type I errors while yielding consistent results under a more liberal IV assumption (Hartwig et al., 2017). The MR‐PRESSO global test and MR–Egger regression were utilized as the main methods to account for horizontal pleiotropy, employing a significance threshold of *p* <.05 (Bowden et al., 2015; Verbanck et al., 2018). In addition, the Cochran's *Q* statistic was used to evaluate the variation across genetic variations and the differences between the IVW and MR–Egger techniques (Greco et al., 2015). A *P* value below.05 indicates considerable heterogeneity. We conducted a leave‐one‐out (LOO) study to evaluate the robustness of the results. This method involves removing each SNP one at a time and then doing MR analysis to determine if the results are significantly influenced by a single SNP (Pierce & Burgess, 2013). In addition, we took steps to reduce the possibility of incorrect findings in all MR investigations by accounting for the false discovery rate (FDR). If there were no IDPs that were statistically significant at the FDR *p*‐value threshold of less than.05, this requirement was relaxed to a *p*‐value of less than.2 (Ma et al., 2023). Following that, we employed the MR Steiger test to assess the probability of reverse causation within our bidirectional MR framework.

In conclusion, our comprehensive assessment of IDPs for their potential causative link with epilepsy was guided by many criteria. (1) The primary analysis has a significant *p*‐value (*p* < .05, determined from IVW) and a *p*_fdr_ of < .2 (**C**.**Wang et al**., 2023). (2) All four MR techniques showed uniform direction and magnitude. (3) The MR results were devoid of heterogeneity or horizontal pleiotropy. (4) Individual SNPs had little effect on the MR estimates.

### 2.5 Replication studies and meta‐analyses

To comprehensively test the robustness of the identified candidate IDPs against the required criteria, a repeat IVW analysis was done with an alternate epilepsy cohort. Initially, GWAS data with the accession number ieu‐b‐8 were examined, followed by a secondary study using GWAS data designated as G6_EPILEPSY (Finngen_R10). We definitively pinpointed the IDPs that are causally linked to epilepsy by conducting a meta‐analysis of the findings from these two MR studies.

### 2.6 Analyzing confounders, multivariable, and subtypes MR studies

We conducted thorough sensitivity analyses to examine the possible presence of horizontal pleiotropy inside our MR framework, identifying any SNPs that may contradict the principles of MR. The studies were performed using the LDtrait tool available on LDlink (https://ldlink.nih.gov/?tab = ldtrait). We examined the connections between each SNP and well‐known risk factors for epilepsy, such as glioma (Smith et al., 2021), educational level (Smith et al., 2021), neuroticism (Zhao et al., 2021), diastolic and systolic blood pressure (Surendran et al., 2020), asthma (van der Meer et al., 2021), and type 2 diabetes (van der Meer et al., 2021). SNPs that showed significant connections with these parameters (*p* < 5 × 10^−6^) were removed, and the MR analysis was repeated to validate our results.

In order to reduce the likelihood of IV violations associated with assumptions 2 and 3 of MR, it is essential to verify that genetic variants are solely associated with a single risk factor. Pleiotropy, which refers to the phenomenon where genetic variants influence several risk variables, is frequently observed (Sanderson, 2021). In order to tackle this issue, we utilized MVMR, which enables the control of genetic variant interactions across different exposures by taking into account numerous correlated exposures (Sanderson, 2021). MVMR stands out by precisely measuring the specific impacts of individual exposures on the outcome, as opposed to univariate MR, which evaluates the overall impact of an exposure on an outcome (Sanderson, 2021). Within our MVMR analysis, we utilized the IVW approach to account for the interactions among the detected IDPs (Burgess & Thompson, 2015; Yun et al., 2023). In MVMR, the IVW strategy includes regressing all exposure‐related SNPs against the outcome, with the outcome's inverse variance serving as weights (Yun et al., 2023).

To gain a clearer understanding of how these identified IDPs relate to epilepsy, we performed a bidirectional MR analysis to investigate the relationship between the IDPs identified in all epilepsies and the 15 specific subtypes of epilepsy.

## 3 RESULTS

### 3.1 Initial analysis

**FIGURE 2:**
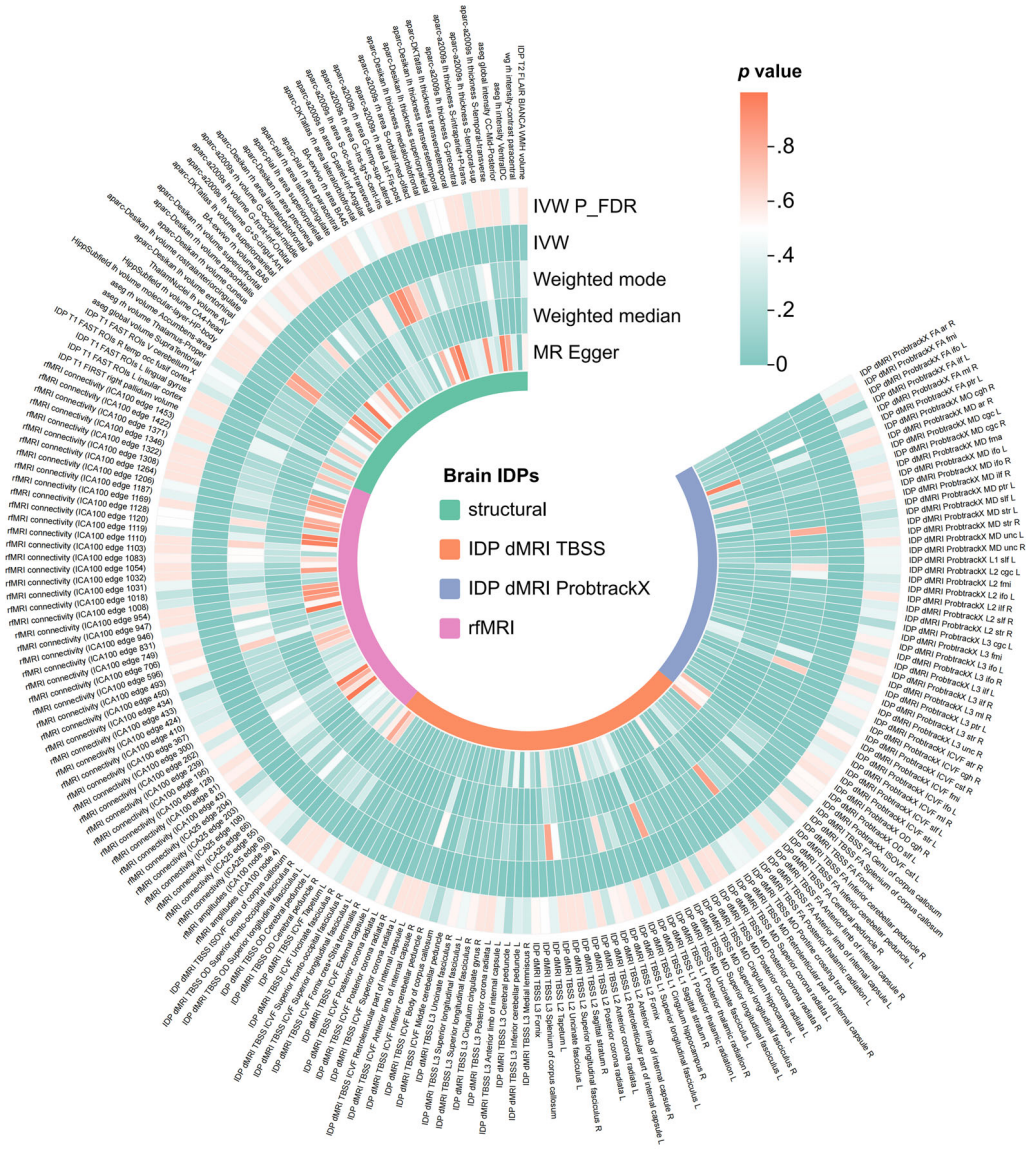
Correlation between brain imaging‐derived phenotypes (IDPs) and all epilepsies. This figure displays circular plots illustrating significant correlations (*p* <.05 for inverse variance weighted [IVW] methods, without heterogeneity or pleiotropy) among 206 IDPs and epilepsy. The outermost circle lists the IDPs, followed by four circles showing *p*‐values from various Mendelian randomization (MR) methods, color‐coded red for nonsignificant and green for significant results. The innermost circle classifies the IDPs. FA, fractional anisotropy; FDR, false discovery rate; dMRI, diffusion MRI; TBSS, Tract‐Based Spatial Statistics; ICVF, intracellular volume fraction; ISOVF, isotropic or free water volume fraction; L1, axial diffusivity; MD, mean diffusivity; OD, orientation dispersion index; rfMRI, resting‐state functional MRI; R/L, right/left; WMH, white matter hyperintensities.

**FIGURE 3:**
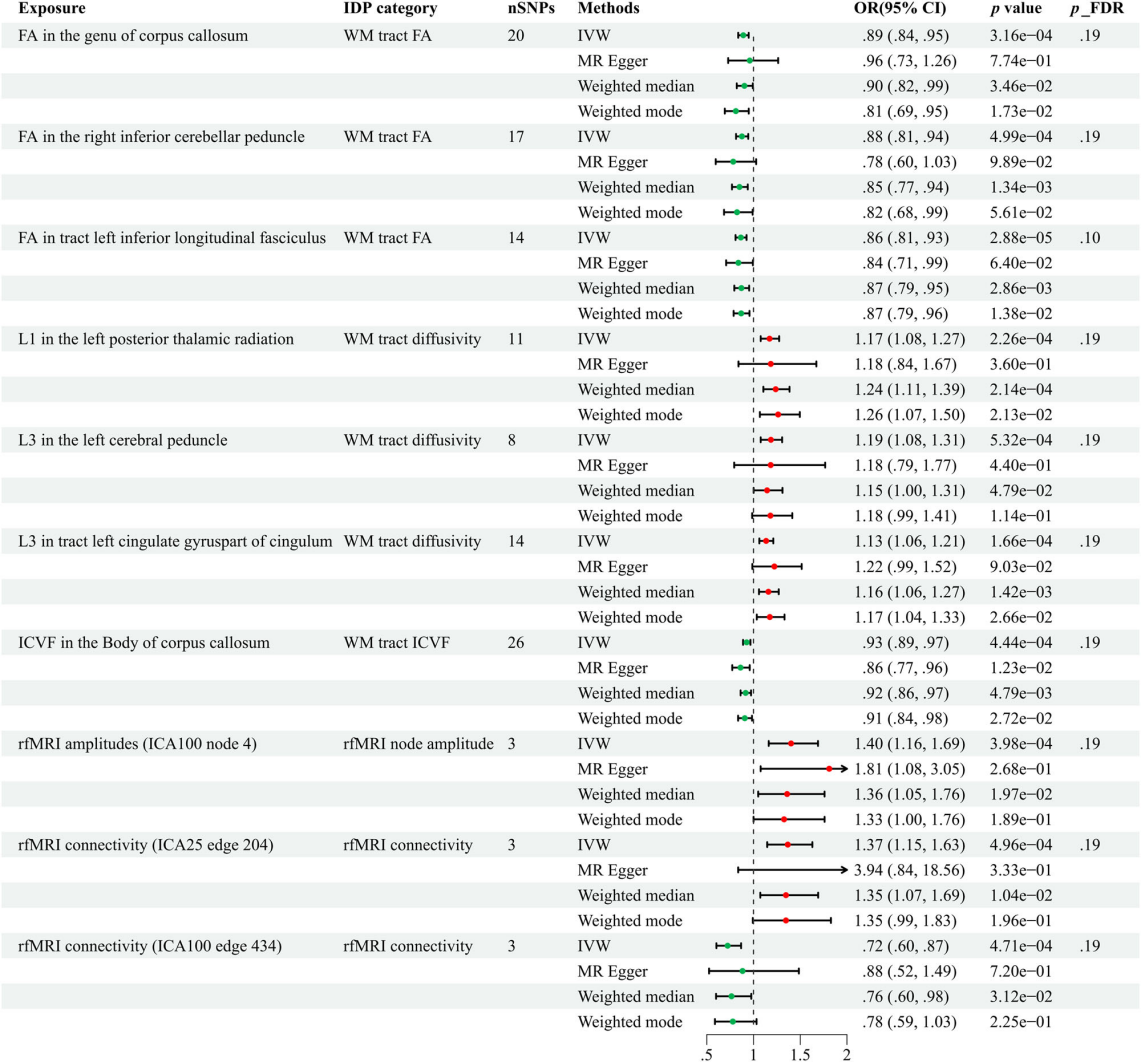
Forest plots were utilized to depict the causal connections between imaging‐derived phenotypes (IDPs) and all epilepsies through different methods. CI, confidence interval; FA, fractional anisotropy; FDR, false discovery rate; fMRI, functional magnetic resonance imaging; ICVF, intracellular volume fraction; IVW, inverse variance weighting; L1, axial diffusivity; L3, radial diffusivity; SNPs, single nucleotide polymorphisms; WM, white matter.

To investigate the possible causal links between IDPs and epilepsy, we employed a two‐sample MR analysis. Preliminary analysis indicated that there may be a causal relationship between 206 IDPs and epilepsy (IVW *p* < .05, with no heterogeneity or pleiotropy), as shown in Figure 2 and Table S2. Nonetheless, after applying the FDR technique to account for multiple testing, successive modifications failed to discover any IDPs that were statistically significant at the PFDR level of 0.05. Using a relaxed significance level of PFDR of 0.20, 10 IDPs emerged as strongly associated with epilepsy, as shown in Figure 3. Seven of these IDPs were derived from dMRI, while three were derived from rfMRI. In the sensitivity analyses, heterogeneity and pleiotropy were not detected, as evidenced by Table 2. The results of the LOO analysis demonstrated that the inclusion of a single SNP did not introduce any bias into the MR estimation (refer to Figure S1). In accordance with the 10 IDPs who satisfied our predetermined screening criteria, they were qualified to advance to the subsequent phase of our inquiry, as further validation by means of complementarity and sensitivity analyses confirmed. Reverse MR analysis did not identify a statistically significant causal relationship among the 10 IDPs (Table S4).

**TABLE 2:** Assessment of heterogeneity and pleiotropy.

Exposure	IDP category	Heterogeneity		Pleiotropy		MR‐PRESSO global test p
		Q	*p*	egger_intercept	*p*	
FA in the genu of corpus callosum	WM tract FA	18.37	.50	0.00	.59	.52
FA in the right inferior cerebellar peduncle	WM tract FA	19.28	.25	0.01	.41	.27
FA in tract left inferior longitudinal fasciculus	WM tract FA	15.60	.27	0.00	.70	.36
L1 in the left posterior thalamic radiation	WM tract diffusivity	10.84	.37	0.00	.95	.41
L3 in the left cerebral peduncle	WM tract diffusivity	6.91	.44	0.00	.99	.49
L3 in tract left cingulate gyrus part of cingulum	WM tract diffusivity	8.09	.84	−0.01	.48	.85
ICVF in the body of corpus callosum	WM tract ICVF	21.53	.66	0.01	.16	.64
rfMRI amplitudes (ICA100 node 4)	rfMRI node amplitude	1.29	.52	−0.02	.49	NA
rfMRI connectivity (ICA25 edge 204)	rfMRI connectivity	1.97	.37	−0.05	.41	NA
rfMRI connectivity (ICA100 edge 434)	rfMRI connectivity	1.41	.49	−0.01	.57	NA

Abbreviations: FA, fractional anisotropy; fMRI, functional magnetic resonance imaging; ICVF, intra‐cellular volume fraction; IDP, imaging‐derived phenotypes; MRPRESSO, Mendelian Randomization Pleiotropy RESidual Sum and Outlier; NA, not available; Q, Cochran's Q statistic; WM, white matter.

### 3.2 Repeated studies and meta‐analysis

**FIGURE 4:**
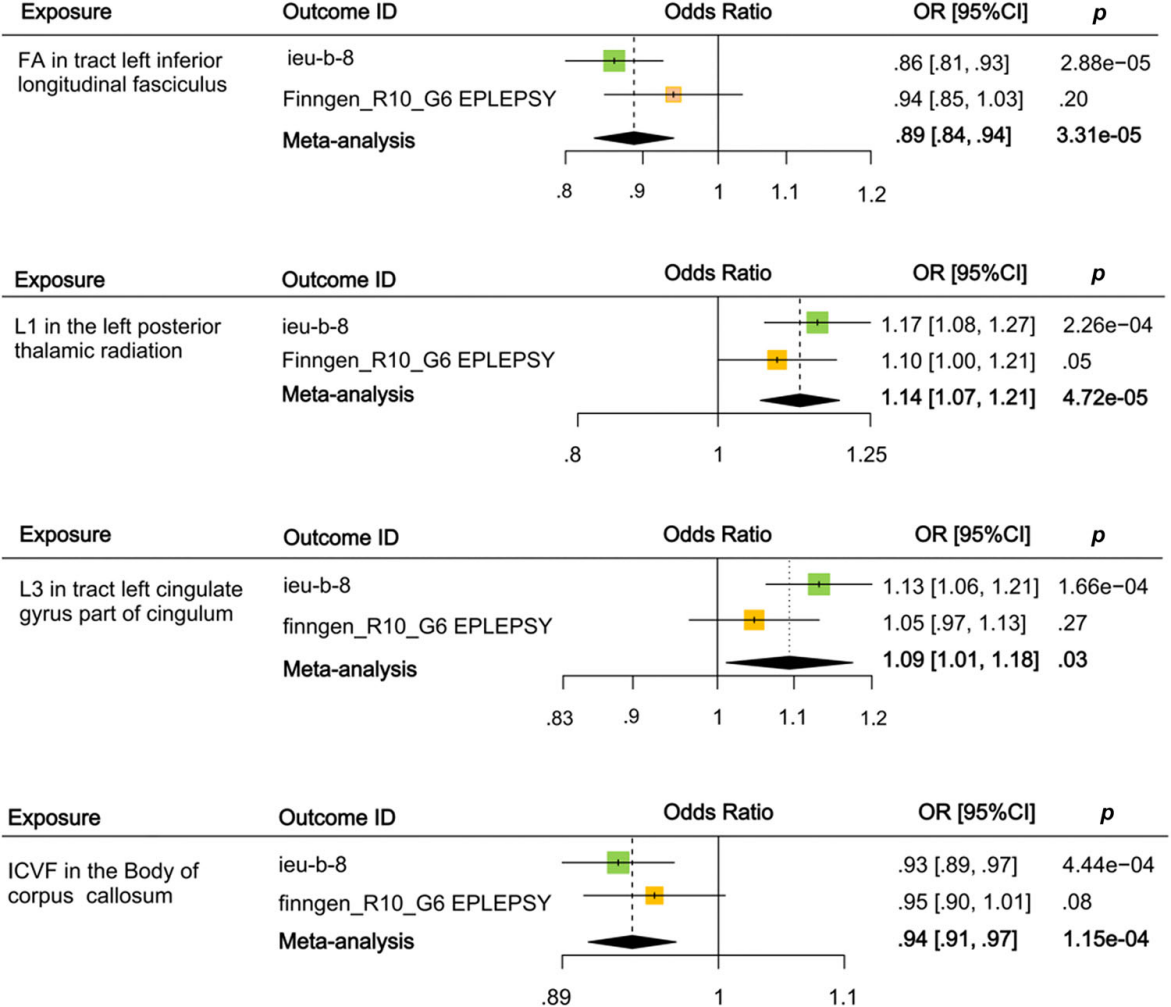
Meta‐analysis of significantly associated (inverse variance weighting (IVW)‐derived *p* <.05) between brain imaging‐derived phenotypes and all epilepsies. CI, confidence interval; FA, fractional anisotropy; ICVF, intracellular volume fraction; IDPs, imaging‐derived phenotypes; L1, axial diffusivity; L3, radial diffusivity; OR, odds ratio.

To bolster the reliability of our estimates, we conducted an MR replication analysis on epilepsy using GWAS data obtained from the FinnGen database (detailed results are presented in Table S5). However, certain results may lack significance due to variances in study demographics and sample sizes. The next meta‐analysis thoroughly identified four independent variables indicating a statistically significant causal connection with epilepsy (see Figure 4 for a full visual representation). Specifically, a noteworthy correlation was observed between a one‐standard deviation (SD) increase in fractional anisotropy (FA) values in the left inferior longitudinal fasciculus (ILF) and an 11% decrease in the risk of epilepsy (odds ratio [OR] = 0.89, 95% confidence interval [CI]: 0.84–0.94, *p* = 3.31×10^−5^). Additionally, there was a 14% correlation between an increase in the mean L1 value on the FA skeleton in the left posterior thalamic radiation and the risk of developing epilepsy (OR = 1.14, 95% CI: 1.07–1.21, *p* = 4.72×10^−5^). Additionally, a 9% elevating risk of epilepsy was associated with each one SD increase in weighted mean L3 in the left cingulate gyrus portion of the cingulum (OR = 1.09, 95% CI: 1.01–1.18, *p* = .03). On the contrary, a one SD increase in the mean intracellular volume fraction (ICVF) within the corpus callosum body observed on the FA skeleton was linked to a 6% decrease in the risk of developing epilepsy (OR = 0.94, 95% CI: 0.91–0.97, *p* = 1.15×10^−4^). Six additional prospective IDPs were omitted from consideration as a result of the meta‐analysis's estimates failing to achieve statistical significance.

### 3.3 Analysis of confounding, MVMR, and subtypes

In this analysis, we systematically eliminated SNPs that could have broken the assumptions of the instrumental variable by conducting thorough sensitivity tests. This assured compliance with the crucial exclusion condition that mandates IVs to be independent of confounders. By utilizing the LDtrait tool, we have confirmed that the SNPs related to each of the four IDPs are not connected to common risk factors for epilepsy, such as glioma, education, neuroticism, diastolic and systolic blood pressure, asthma, and type 2 diabetes. Our investigation has revealed seven SNPs located within the IVs of the four IDPs that are connected with these factors. The specific details can be found in Table S7. Our findings remained statistically significant even after these SNPs were omitted, thereby establishing the robustness of our results. Significantly, there was a correlation observed between the FA in the left inferior longitudinal fasciculus (OR = 0.88, 95%CI: 0.82–0.94, *p* = 8.16×10^−5^), the mean L1 on the FA skeleton in the left posterior thalamic radiation (OR = 1.17, 95% CI: 1.08–1.27, *p *= 2.26×10^−4^), the weighted‐mean L3 in the left cingulate gyrus portion of the cingulum (OR = 1.14, 95% CI: 1.07–1.22, *p* = 1.08×10^−4^), and the mean ICVF in the body of the corpus callosum on the FA skeleton (OR = 0.92, 95% CI: 0.88–0.96, *p* = 3.00×10^−4^)(Table S8).

**FIGURE 5:**
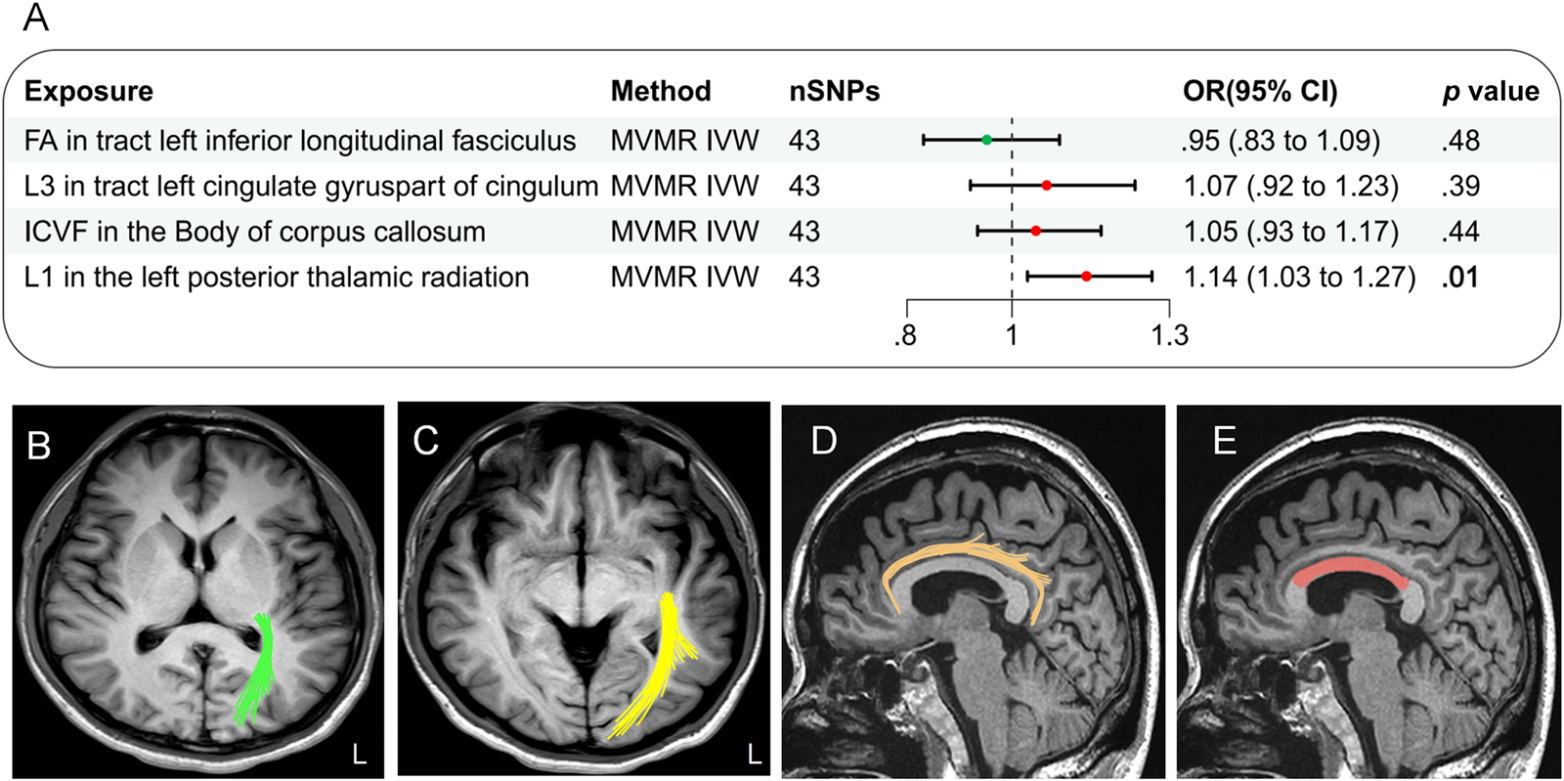
(a) Multivariable Mendelian randomization (MR) analysis of the final identified imaging‐derived phenotypes (IDPs); (b) left posterior thalamic radiation (green) in the transverse axial position as depicted in the T1WI schematic; (c) the left inferior longitudinal fasciculus (yellow) is illustrated schematically in the transverse axial position on T1WI; (d) sagittal representation of the left cingulate bundle (orange) as depicted in the T1WI schematic; (e) schematic of the body of corpus callosum (red) in sagittal position on T1WI. CI, confidence interval; FA, fractional anisotropy; ICVF, intracellular volume fraction; IVW, inverse variance weighted; L1, axial diffusivity; L3, radial diffusivity; MVMR, multivariable Mendelian randomization; OR, odds ratio; SNP, single nucleotide polymorphism; T1WI, T1 weighted imaging.

Additional modifications to account for interactions among IDPs, such as the IVW method and MVMR (illustrated in Figure 5A), provided support for the hypothesis that the mean L1 in the left posterior thalamic radiation on FA (Figure 5B), which was predicted genetically, might have an independent causal relationship with epilepsy, separate from other IDPs. Figure 5 illustrates the schematic diagrams for the additional three IDPS: the left inferior longitudinal fasciculus (Figure 5C), the left cingulate gyrus portion of the cingulum (Figure 5D), and the body of the corpus callosum (Figure 5E).

**FIGURE 6:**
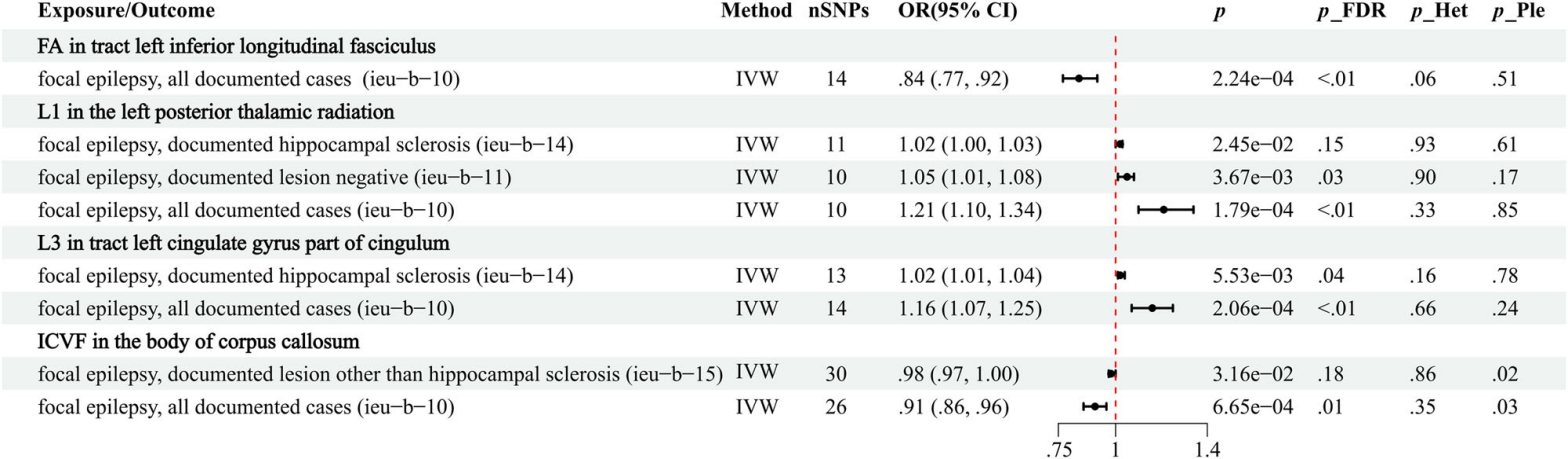
A forest plot analyzing epilepsy subtypes, highlighting the significant associations between imaging‐derived phenotypes (IDPs) identified in relation to all epilepsies and the various epilepsy subtypes. CI, confidence interval; FA, fractional anisotropy; FDR, false discovery rate; Het, heterogeneity; ICVF, intracellular volume fraction; IVW, inverse variance weighted; L1, axial diffusivity; L3, radial diffusivity; OR, odds ratio; Ple, Pleiotropy; SNP, single nucleotide polymorphism.

We performed bidirectional MR analyses on the four IDPs associated with all epilepsies, identified in the previous analysis, against 15 other epilepsy subtypes (results are shown in Table S9). Our findings indicate that these IDPs are predominantly linked to focal epilepsy. Specifically, L1 in the left posterior thalamic radiation is positively associated with the risk of hippocampal sclerosis and documented lesion negative focal epilepsy, while L3 in the tract of the left cingulate gyrus part of the cingulum is positively associated with the risk of hippocampal sclerosis‐related focal epilepsy. Sensitivity analyses revealed pleiotropy between ICVF in the body of the corpus callosum and two epilepsy subtypes, whereas no heterogeneity or pleiotropy was detected between the remaining IDPs and the epilepsy subtypes (Figure 6). Additionally, reverse MR analyses did not demonstrate any causal relationship between the epilepsy subtypes and these four IDPs (Table S10).

## 4 DISCUSSION

Observational studies have previously highlighted correlations between epilepsy and IDPs, yet the causative nature of these associations remains uncertain. In this study, we utilized a robust MR framework to examine potential causal relationships between epilepsy and 3935 IDPs, drawing on data from two extensive GWAS. Our analysis identified four IDPs with statistically significant evidence of a potential causal influence on all epilepsies. Further subtype analysis revealed that three of these IDPs are primarily associated with FE. Specifically, L1 in the left posterior thalamic radiation was found to be positively associated with an increased risk of hippocampal sclerosis (HS) and lesion‐negative FE, while L3 in the tract of the left cingulate gyrus, part of the cingulum, was positively associated with an increased risk of hippocampal sclerosis‐related focal epilepsy (HS‐FE).

In our study, we found that the risk of developing epilepsy, particularly FE, is associated with decreased FA in the tract of the ILF. The ILF connects the occipital and temporal lobes via a white matter pathway (Shin et al., 2019). FA is often understood as a measure of fiber density, axon diameter, and myelination in the brain's white matter (WM), with decreased FA values indicating axonal damage (Y. Zhang et al., 2023). Temporal lobe epilepsy is the most common form of FE, and previous studies have observed reduced FA values in the ILF of temporal lobe epilepsy patients (Li et al., 2019; Y. Zhang et al., 2023). Our research further provides causal evidence supporting these previous findings, indicating that decreased FA in the ILF is significantly associated with the occurrence of epilepsy.

Our study revealed a significant positive correlation between epilepsy risk and the L1 value (axial diffusivity) in the left PTR, which was found to be direct and independent. Subtype analysis indicated that this association is particularly related to HS‐FE and lesion‐negative FE. The PTR, as a crucial neural pathway connecting the occipital and parietal lobes with the thalamus, plays a vital role in maintaining normal neural transmission and brain function (Kamali et al., 2023). The observed increase in the L1 value suggests potential axonal damage or dysfunction within the PTR, which may facilitate the spread and maintenance of epileptic discharges (Wheeler‐Kingshott & Cercignani, 2009; Y. Zhang et al., 2023). This finding aligns with previous studies. For example, Sundram et al. (2010) reported a significant reduction in white matter fibers in the posterior thalamic radiation of TLE patients with psychiatric symptoms, underscoring the role of PTR in epilepsy. Furthermore, our study found that the increased L1 value is associated with HS‐FE, a common cause of adult epilepsy, which is characterized by the loss of hippocampal neurons, gliosis, and synaptic reorganization (Vinti et al., 2021). These pathological changes not only affect the hippocampus but may also impact broader brain regions through pathways like the PTR (Urquia‐Osorio et al., 2022). Additionally, we observed an increase in L1 value in patients with negative lesion FE. This suggests that even in the absence of visible structural lesions, microstructural damage or abnormalities in neural fiber tracts may contribute to epilepsy (Goubran et al., 2015). This highlights the importance of advanced neuroimaging techniques in identifying the underlying pathological mechanisms of epilepsy and provides new insights for the precise diagnosis and treatment of epilepsy.

Our study identified a significant association between increased L3 value (radial diffusivity, [RD]) in the left cingulum and elevated epilepsy risk, particularly in FE and HS‐FE. The cingulum, the longest C‐shaped marginal pathway, is a crucial component of the Papez circuit (Kamali et al., 2023). It facilitates the connection between prefrontal and parietal projections and the hippocampus located in the parahippocampal gyrus of the temporal lobe (Kamali et al., 2023). An increase in L3 value may reflect microstructural changes within the neural fibers of the cingulum, especially potential myelin damage, which increases susceptibility to epilepsy (Wheeler‐Kingshott & Cercignani, 2009). Our findings align with previous studies indicating a relationship between the cingulum and epilepsy (Campos et al., 2015; Sundram et al., 2010). For instance, Campos et al. (2015) reported increased ipsilateral RD in the cingulum of patients with temporal lobe epilepsy with hippocampal sclerosis (TLE‐HS) and focal cortical dysplasia (FLE‐FCD). These results collectively underscore the close link between structural changes in the cingulum and the occurrence of epileptic seizures.

In our study, we identified a significant association between decreased intracellular volume fraction (ICVF) in the corpus callosum—the largest axonal bundle connecting the left and right cortical hemispheres (Lynton; Suárez et al., 2023)—and an increased risk of all epilepsies. Previous studies have shown that the corpus callosum is a major pathway for seizure propagation in patients with generalized epilepsy (Chen et al., 2020). Additionally, the rapid spread of seizures in FE is facilitated by the corpus callosum (Chen et al., 2020). ICVF, a neuroimaging‐derived metric, reflects neural fiber density and orientation dispersion (H. Zhang et al., 2012). Sone et al. (2018) reported a reduction in ICVF in the ipsilateral temporal pole of patients with TLE. Despite these findings, research specifically exploring the relationship between corpus callosum ICVF and epilepsy remains limited. Furthermore, our subtype analysis revealed that ICVF exhibits pleiotropy in its association with FE, and no causal relationships with other identified subtypes were observed. These findings suggest that further investigation into the role of ICVF alterations in the corpus callosum is warranted to better understand their implications in epilepsy.

Although our findings align with some previous research, we did not replicate certain associations between brain phenotypes and epilepsy reported in earlier studies. For instance, while numerous studies have linked epilepsy to HS (Campos et al., 2015; Middlebrooks et al., 2024; Urquia‐Osorio et al., 2022), this association was not observed in our investigation. Several factors may explain these discrepancies. Initially, in our discovery cohort, we identified a potential causal relationship between two IDPs related to the hippocampal cingulum and all epilepsies. However, this association was not significant after applying FDR correction (see in Figure 2 and Table S2), suggesting that the initial findings may have been false positives or that the true effect sizes are too small to be detected with our MR approach. Additionally, previous observational studies often employed smaller sample sizes, which may not have adequately controlled for unknown confounding factors. In contrast, our study uses MR, which leverages genetic polymorphisms as proxies for causal inference, reducing the impact of confounding and reverse causation (Skrivankova et al., 2021). Furthermore, variations in study populations, imaging techniques, and analytical methods could contribute to inconsistencies in findings. The classification of epilepsy and its subtypes is also complex and frequently revised, which may add to the variability observed across studies (Thijs et al., 2019; Whelan et al., 2018).

This study has several limitations. First, the majority of participants were of European descent, which may impact the generalizability of our findings to other ethnicities. Second, due to limited available IVs, we employed less strict criteria for their selection. Despite this, the robustness of our IVs is confirmed, as all selected SNPs showed F‐statistics above 10. Additionally, the consistency in causal direction, as demonstrated by the Steiger test, lends support to our methodological approach. Additionally, our exploratory study identified four IDPs with potential causal links to epilepsy, which may improve early diagnosis and targeted interventions. However, given epilepsy's multifactorial nature, the MR evidence linking IDPs to epilepsy risk should be interpreted with caution, as it does not rule out other factors. Furthermore, since this inference is based solely on MR analysis, further validation is needed to confirm its relevance to epilepsy etiology. Thus, MR‐derived effect sizes should be cautiously applied in clinical settings (Guo et al., 2022), and rigorous trials and additional research are required before clinical use.

## 5 CONCLUSION

To summarize, this study explored the relationship between genetically determined IDPs and epilepsy risk through MR. The outcomes present genetic evidence suggesting a potential causal link between neuroimaging characteristics and epilepsy. These findings hold promise for advancing epilepsy risk prediction and intervention through the analysis of brain imaging data.

## AUTHOR CONTRIBUTIONS

**Fangyan Li**: Data curation; methodology; writing—original draft; writing—review and editing. **Maowen Tang**: Conceptualization; writing—review and editing. **Cheng Hao**: Visualization; writing—review and editing; data curation. **Menghua Yang**: Data curation; writing—review and editing. **Yue Pan**: Data curation; writing—review and editing. **Pinggui Lei**: Conceptualization; supervision; project administration; writing—review and editing; funding acquisition.

## CONFLICT OF INTEREST STATEMENT

The authors declare no conflicts of interest.

### PEER REVIEW

The peer review history for this article is available at https://publons.com/publon/10.1002/brb3.70051

## Supporting information




**Supplementary Figures 1**: MR leave‐one‐out sensitivity analyses of the causal relationship between 10 IDPs and epilespy.

Table S1 A total of 18,943 SNPs were utilized in the initial analyses to investigate the causal relationship between IDPs and epilepsy.
Table S2 The initial analysis's results of the MR analysis of the IDPS with epilepsy
Table S3 The information of IVs for epilepsy to ten IDPs in forward MR
Table S4 MR analysis of epilepsy with 10 IDPs in inverse analysis.
Table S5 MR results in replication analyses
Table S6 IVs applied to MR replication analysis
Table S7 Confounders identified from LDlink
Table S8 MR analysis results for the four IDPs following the elimination of confounding variables
Table S9 MR results for the analysis of four IDPs with 15 epilepsy subtypes.
Table S10 Inverse MR analysis of 15 epilepsy subtypes with 4 IDPs.
Table S11 An explanation of the IDPS utilized in this research.

## Data Availability

The BIG40 web browser was used to gather GWAS statistics on brain IDPs (https://open.win.ox.ac.uk/ukbiobank/big40). GWAS data pertaining to epilepsy can be accessed through the IUE database (https://gwas.mrcieu.ac.uk/datasets/ieu‐b‐8/) and the  FinnGen database (https://www.finngen.fi/en/access_results).
