# Supplementary material for: Brain imaging traits and epilepsy: Unraveling causal links via mendelian randomization
Source: Brain Behav. 2024 Sep 30;14(10):e70051. doi: 10.1002/brb3.70051 (PMC11442987; doi:10.1002/brb3.70051)
Supplement: Supplementary file 1 — Supplementary Figures 1: MR leave‐one‐out sensitivity analyses of the causal relationship between 10 IDPs and epilespy. [file BRB3-14-e70051-s001.docx]

**Supplementary Figures**

# Brain Imaging Traits and Epilepsy: Unraveling Causal Links via Mendelian Randomization

**Supplementary Figures**

**Supplementary Figures 1**: MR leave-one-out sensitivity analyses of the causal relationship between 10 IDPs and epilespy.

| 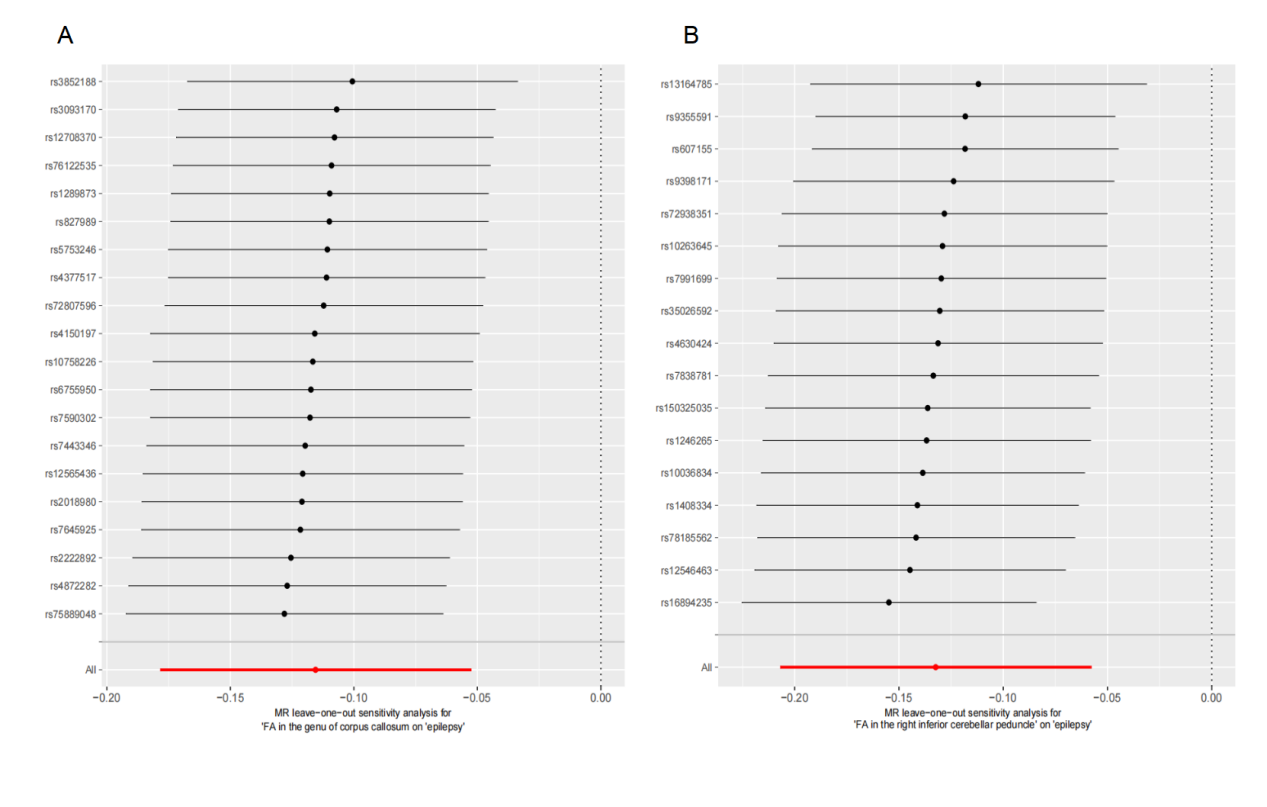  **Figure S1** (A) MR leave-one-out sensitivity analyses of the causal relationship between FA in the genu of corpus callosum and epilepsy；(B) MR leave-one-out sensitivity analyses of the causal relationship between FA in the right inferior cerebellar peduncle and epilepsy. MR, Mendelian randomization; FA, fractional Anisotropy. |
| --- |

| 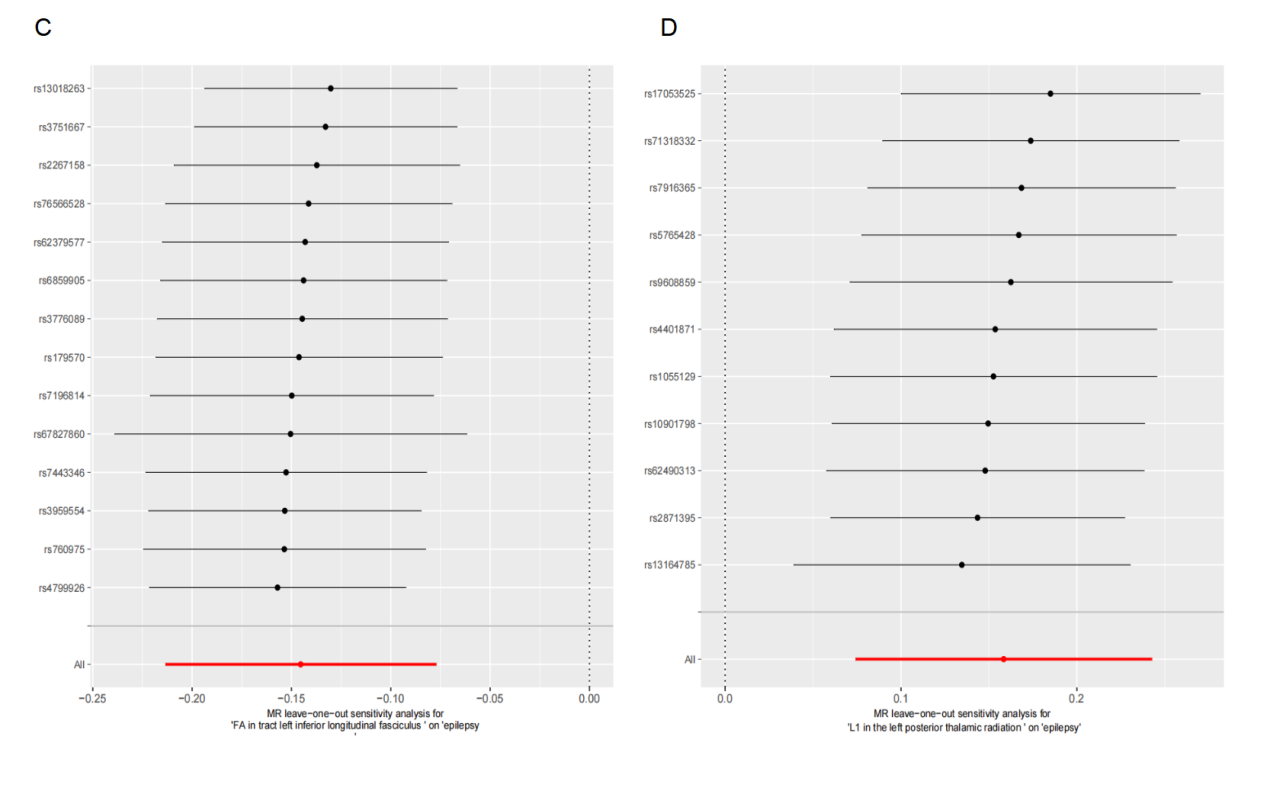  **Figure S1** (C) MR leave-one-out sensitivity analyses of the causal relationship between FA in tract left inferior longitudinal fasciculus and epilepsy；(D) MR leave-one-out sensitivity analyses of the causal relationship between L1 in the left posterior thalamic radiation and epilepsy. MR, Mendelian randomization; FA, fractional Anisotropy. |
| --- |

| 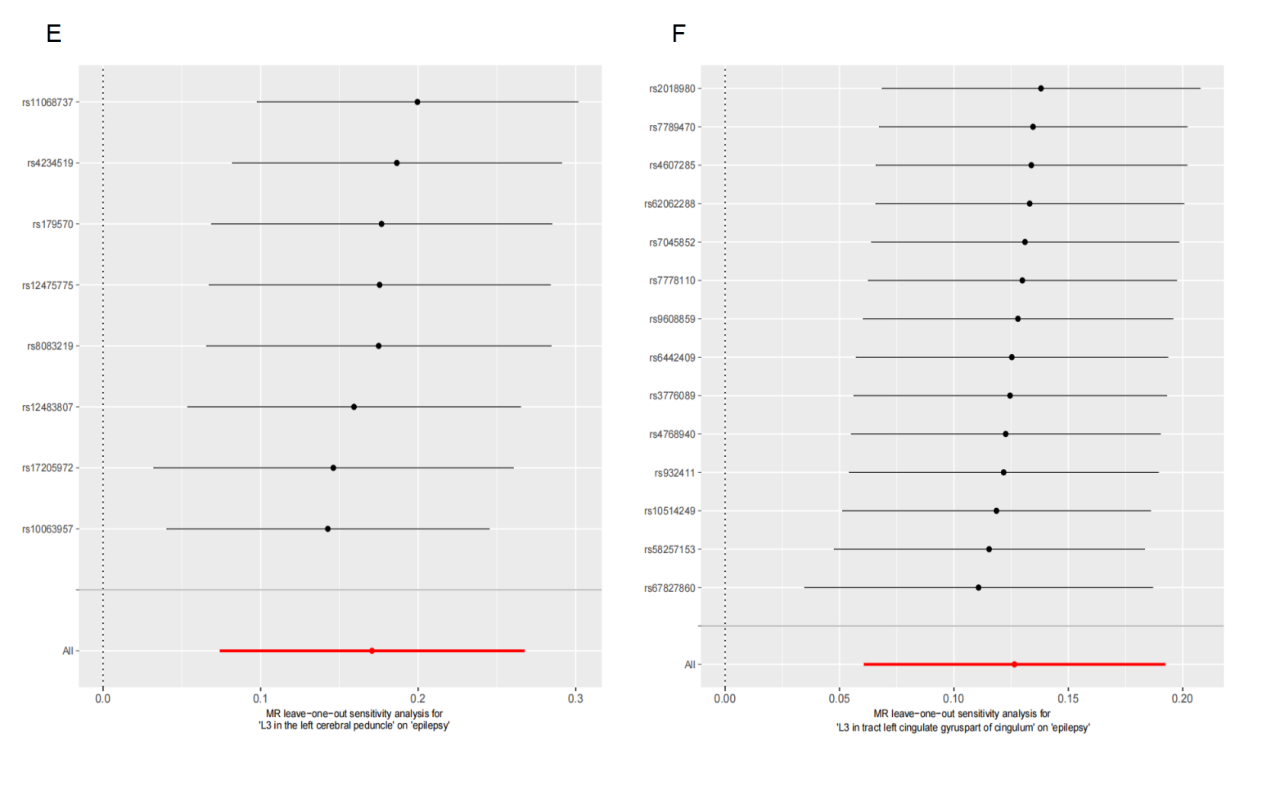  **Figure S1** (E) MR leave-one-out sensitivity analyses of the causal relationship between L3 in the left cerebral peduncle and epilepsy；(F) MR leave-one-out sensitivity analyses of the causal relationship between L3 in tract left cingulate gyruspart of cingulum and epilepsy. MR, Mendelian randomization. |
| --- |

| 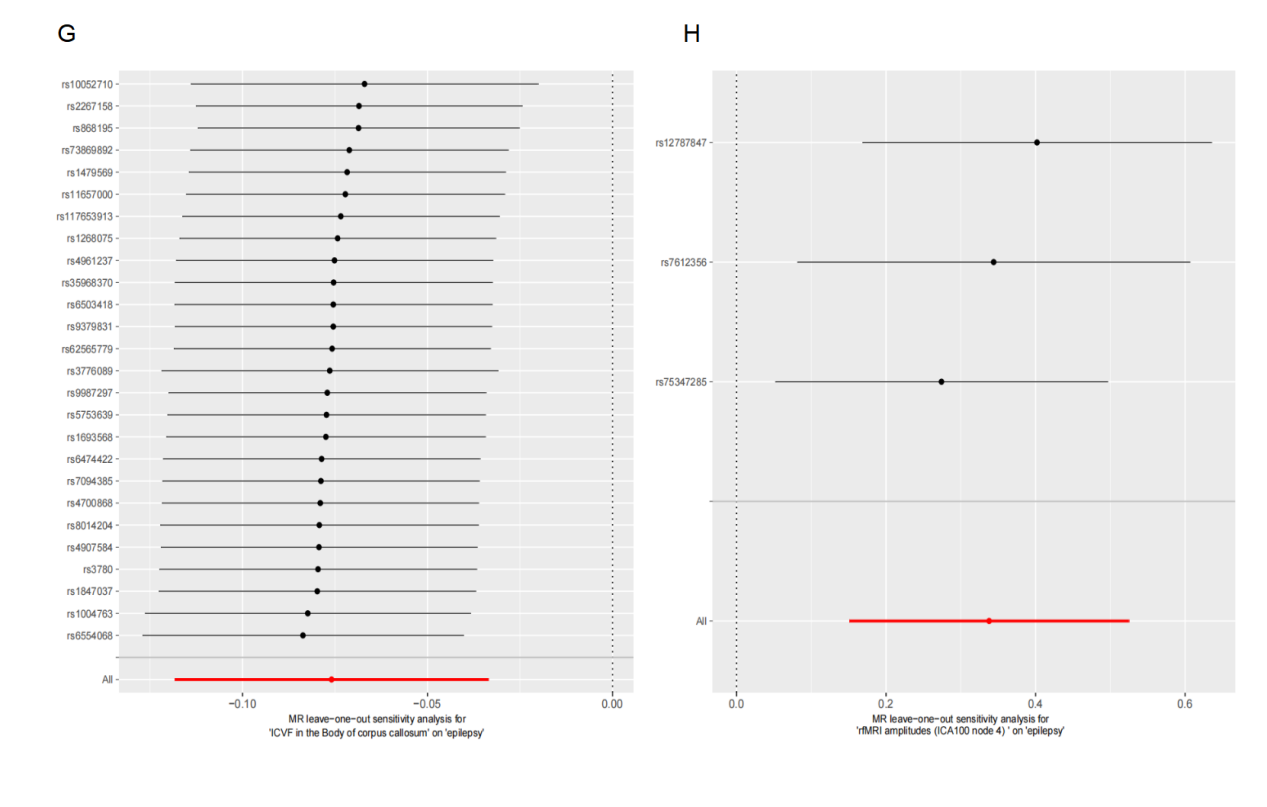  **Figure S1** (G) MR leave-one-out sensitivity analyses of the causal relationship between ICVF in the Body of corpus callosum and epilepsy；(H)MR leave-one-out sensitivity analyses of the causal relationship between rfMRI amplitudes (ICA100 node 4) and epilepsy. MR, Mendelian randomization; ICVF, intracellular volume fraction. |
| --- |
| 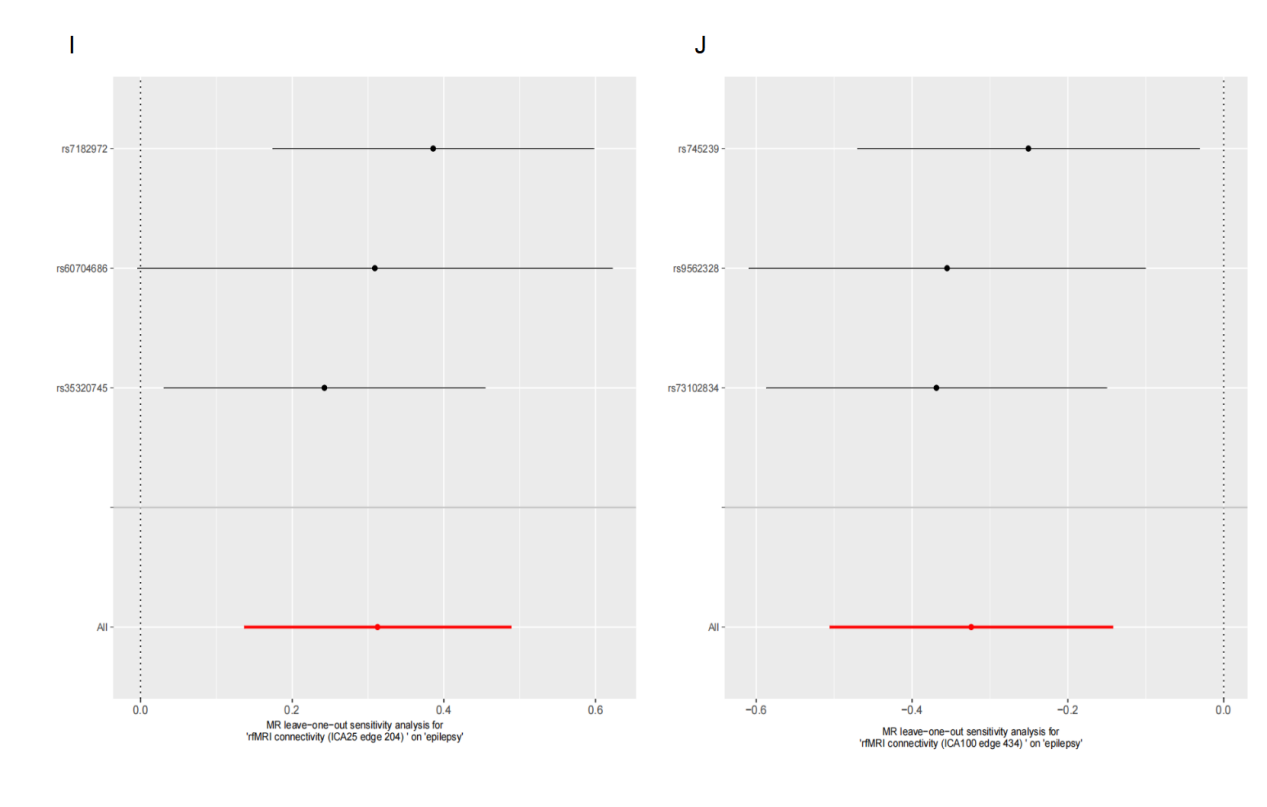  **Figure S1** (I) MR leave-one-out sensitivity analyses of the causal relationship between rfMRI connectivity (ICA25 edge 204) and epilepsy；(J) MR leave-one-out sensitivity analyses of the causal relationship between rfMRI connectivity (ICA100 edge 434) and epilepsy. MR, Mendelian randomization. |
